# Motivators and demotivators to accessing malaria in pregnancy interventions in sub-Saharan Africa: a meta-ethnographic review

**DOI:** 10.1186/s12936-022-04205-7

**Published:** 2022-06-03

**Authors:** Matilda Aberese-Ako, Phidelia Doegah, Evelyn Acquah, Pascal Magnussen, Evelyn Ansah, Gifty Ampofo, Dominic Dankwah Agyei, Desmond Klu, Elsie Mottey, Julie Balen, Safiatou Doumbo, Wilfred Mbacham, Ouma Gaye, Margaret Gyapong, Seth Owusu-Agyei, Harry Tagbor

**Affiliations:** 1 University of Health and Allied Sciences, Ho, Volta Region Ghana; 2 Faculty of Health and Medical Sciences, Centre for Medical Parasitology, University of Copenhagen, Copenhagen, Denmark; 3 School of Health and Related Research, The University of Sheffield, Sheffield, UK; 4 University of Sciences, Techniques and Technologies of Bamako, Malaria Research and Training Center, Bamako, Mali; 5 The Fobang Institutes for Innovations in Science and Technologies & The Biotechnology Center, The Centre for Health Innovations and Translational Research, University of Yaounde I, Yaounde, Cameroon; 6 Faculty of Medicine, University Cheikh Anta Diop Dakar, Dakar, Senegal

**Keywords:** Malaria in pregnancy, De(motivators), Community, Socio-cultural, Sub-Saharan Africa, Health system, Environment, Interventions, Individual

## Abstract

**Background:**

Despite the introduction of efficacious interventions for malaria control, sub-Saharan Africa continues to bear the highest burden of malaria and its associated effects on vulnerable populations, such as pregnant women and children. This meta-ethnographic review contributes to literature on malaria in pregnancy interventions in sub-Saharan Africa by offering insights into the multiple factors that motivate or demotivate women from accessing MiP interventions.

**Methods:**

A meta-ethnographic approach was used for the synthesis. Original qualitative research articles published from 2010 to November 2021 in English in sub-Saharan Africa were searched for. Articles focusing on WHO’s recommended interventions such as intermittent preventive treatment with sulfadoxine-pyrimethamine, long-lasting insecticidal nets and testing and treatment of Malaria in Pregnancy (MiP) were included. Selected articles were uploaded into Nvivo 11 for thematic coding and synthesis.

**Results:**

Twenty-seven original qualitative research articles were included in the analysis. Main factors motivating uptake of MiP interventions were: (1) well organized ANC, positive attitudes of health workers and availability of MiP services; (2) Women’s knowledge of the effects of malaria in pregnancy, previous experience of accessing responsive ANC; (3) financial resources and encouragement from partners, relatives and friends and (4) favourable weather condition and nearness to a health facility. Factors that demotivated women from using MiP services were: (1) stock-outs, ANC charges and health providers failure to provide women with ample education on the need for MiP care; (2) perception of not being at risk and the culture of self-medication; (3) fear of being bewitched if pregnancy was noticed early, women’s lack of decision-making power and dependence on traditional remedies and (4) warm weather, long distances to health facilities and the style of construction of houses making it difficult to hang LLINs.

**Conclusions:**

Health system gaps need to be strengthened in order to ensure that MiP interventions become accessible to women. Additionally, health managers need to involve communities in planning, designing and implementing malaria interventions for pregnant women. It is important that the health system engage extensively with communities to facilitate pregnant women and communities understanding of MiP interventions and the need to support pregnant women to access them.

## Background

Globally, an estimated 241 million malaria cases were recorded in 85 malaria endemic countries in 2020. This was an increase from 229 million cases recorded in 2019 in 87 malaria endemic countries [1–3]. Most of the increase was recorded in the World Health Organization (WHO) African region with an estimated 228 million cases in 2020, which accounted for about 95% of the cases, with the most affected in the population being pregnant women and children [1]. Consequently, the highest malaria in pregnancy cases globally were recorded within the African region [2, 3]. In 2020, of the 33 moderate to high transmission countries in the WHO African Region, 11.6 million (34%) of the estimated 33.8 million pregnancies were exposed to malaria infection during pregnancy [1]. The highest prevalence of exposure to malaria during pregnancy (39.8%), was recorded in West Africa followed by Central Africa (39.4%), while East and Southern Africa recorded a prevalence of 22% [1].

In order to avert the negative consequences of malaria in pregnancy, such as low birth weight and mortality, most sub-Saharan African countries have implemented the WHO recommended interventions for Malaria in Pregnancy (MiP) as a component of maternal health care [2, 4–9]. This entails, in combination with vector control, the prompt diagnosis and effective treatment of malaria in pregnancy, the use of intermittent preventive treatment with sulfadoxine-pyrimethamine (IPTp-SP) as part of ANC, and the distribution, adherence and appropriate use of long-lasting insecticidal nets (LLINs) during pregnancy [10–12]. IPTp-SP is given at scheduled periods except during the first trimester of pregnancy [2, 13, 14], to ensure that a high proportion of women receive at least three doses of SP during pregnancy [13].

Despite these interventions, sub–Saharan African countries did not achieve the reset Abuja targets of 100% pregnant women having access to IPTp and 100% use of LLINs by 2015 [15].^1^ In 2020, the WHO reported that on average, 80% of all pregnant women visited ANC clinics at least once, with 62% receiving at least one dose of IPTp, 49% receiving at least two doses of IPTp and 34% receiving at least three doses of IPTp in the 33 countries with moderate to high transmission of malaria in the African sub-region [2]. Additionally, 52% of pregnant women used LLINs in 2020 [16]. The WHO estimates that MiP resulted in 822,000 children being born with low birth weight, with 49% of them being in the West Africa sub-region [2].

Challenges and gaps in the implementation of MiP interventions in the sub-region have been reported [15, 17–21]. Various studies have been carried out over the years to understand why these interventions have not had the desired impact. Poor organization of health service delivery, confusion between policy makers and implementers over the timing of each IPTp-SP dose, stock-outs, user fees and negative attitudes of health workers have contributed to low utilization of MiP interventions [9, 22–28]. Other challenges such as poverty, certain cultural beliefs and practices, poor antenatal attendance and lack of knowledge on malaria and its effects on pregnancy have equally been reported [9, 18, 23, 29–32]. Systematic and meta-analysis reviews on MiP interventions suggest that barriers to accessing these interventions include high cost of treatment, lack of knowledge of drug safety and self-medication [23, 33]. However, health education and previous experience of antenatal care encouraged women to access MiP interventions [23, 33]. Additionally, a systematic review on IPTp utilization in the sub-Saharan Africa region found that policy gaps, stock-outs, poor management practices and negative staff attitudes contributed to limited access [34].

Only two qualitative reviews on MiP interventions have been identified [35, 36]. Ribera et al. [35] used personal fieldwork experiences and a non-systematic search strategy to develop models for qualitative approaches to research on factors affecting MiP. The first study to use a meta-ethnographic approach to analyse and synthesize qualitative findings on MiP is Pell et al. [36]. The study reported on malaria and risk in pregnancy concepts, attitudes towards interventions, structural factors affecting delivery and uptake, and perceptions of ANC up till April 2010 [36]. The two studies have provided the foundation and insight into qualitative reviews on MiP research studies. Nevertheless, meta-ethnographic review is well established in health research [37–39]. This meta-ethnographic review contributes to the literature on health research, which includes MiP interventions in sub-Saharan Africa from 2010 to November 2021. It offers insights into the multiple factors that motivate women and those that demotivate them from accessing MiP interventions in sub-Saharan Africa.

## Methods

According to Noblit and Hare [40], a meta-ethnography review is “…*an interpretative and not an aggregative process, where there is reciprocal translation of information into one another*.” Thus, this review sought to understand and transfer ideas, concepts and metaphors across the 27 different studies selected for the review [41–43]. Noblit and Hare [40] have recommended seven phases in conducting meta-ethnography, which this study employed as follows: (1) getting started or identifying an area of intellectual interest for a review; (2) deciding what is relevant to the initial interest; (3) reading the studies; (4) determining how the studies are related; (5) translating the studies into one another; (6) synthesizing translations; and (7) expressing the synthesis. Further to this, the study adopted the GRADE-CERQual framework to overcome methodological limitation (the extent to which there are problems in the design or conduct of the primary studies that contributed evidence to a review finding), ensure relevance (the extent to which the body of evidence from the primary studies supporting a review finding is applicable to the context), guarantee coherence (the extent to which the review finding is well grounded in data from the contributing primary studies and provides a convincing explanation for the patterns found in these data), and to determine data adequacy (an overall determination of the degree of richness and quantity of data supporting a review finding) [44].

### Identification of themes/concepts and selection criteria

The review explored qualitative studies reporting on barriers and facilitators to women accessing MiP interventions implemented in sub-Saharan Africa [2]. The study team defined the focus of the synthesis, made decisions on selection criteria and searched and read the literature to understand the topic of interest and to define the focus of the synthesis.

Two major themes or concepts were identified: motivation and demotivation. Motivation in this study is perceived as an “*incitement of the will or a consideration that leads to action*” [45]. It also means an individual’s degree of willingness to exert and maintain an effort towards achieving a goal [46]. Demotivation is derived from the verb “*to demotivate*”, which is perceived as causing a loss of motivation in an individual or in a person [45].

#### Inclusion criteria

The focus of the synthesis included qualitative articles that reported on the three major WHO recommended MiP interventions for sub-Saharan Africa. *Since beliefs, experiences, health care context and social phenomena change over time* [38] and to enhance the relevance of the review, the study period was limited to eleven years (from May 2010 to November 2021). Other criteria were that, the selected studies should have obtained ethical clearance, should be published in English and the full article should be accessible. Also, this study in accordance with more recent debates by Noblit [37], expanded the criteria for meta-ethnography, from articles employing only ethnographic methodological approach to include all studies using other qualitative methodological approaches such as case study, phenomenology and biography. For studies that used mixed methods approach, only the qualitative results were extracted for analysis.

#### Exclusion criteria

All non-English articles, articles published before May 2010, articles that did not report on ethical approval, those that lacked full abstract and clinical trials were excluded.

#### Search and selection of studies

The first search was carried out in 2017 and the second was carried out in November, 2021. Searches were conducted in databases that included PubMed/Medline/PMC and HINARI. Search terms and phrases used included pregnancy, pregnant, community, benefits, motivation, demotivation, social, cultural, socio-cultural, malaria, interventions, women, sub-Saharan Africa, behaviours, attitudes, perceptions, practices, qualitative, community, vulnerable population, barriers, facilitators, IPTp, SP, ITN, LLIN, bed nets, malaria treatment in pregnancy, qualitative, ethnography, phenomenology, case studies and grounded theory.

The articles were subjected to title and abstract screening. All the abstracts of the selected articles that met the criteria for selection were then retrieved and those that directly addressed the themes were regrouped. This was followed by a second search to find the full text of the regrouped articles. The articles were read and those that met the final criteria were 27. They were then uploaded into Nvivo 11 for coding and analysis. Authors, MA and PD read the selected studies to identify concepts of interest and how they were related to each other. Subsequently, open coding was done, which facilitated the identification of two major themes (motivation and demotivation). This was followed by axial coding, where the emerging themes were categorized and regrouped into four sub themes (health system, individual, socio-cultural and environmental), under the two major themes.

MA and PD carried out further reading of the codes, categorized and grouped them into the four sub themes: individual, health system, socio-cultural and environmental factors influencing motivation and demotivation in accessing MiP interventions, with the support of a matrix. The matrix facilitated a better understanding of the meaning, relationships, interactions and how the codes translated into each other. The codes were then interpreted and translated into one another in accordance with meta-ethnography.

## Results

**Fig. 1:**
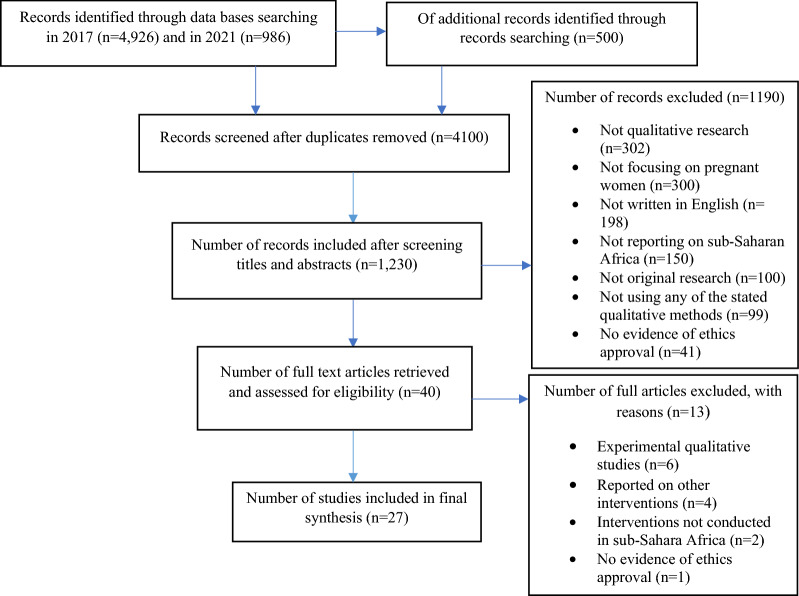
Flowchart of selection of studies. Motivators to utilization Demotivators to utilization

**Fig. 2:**
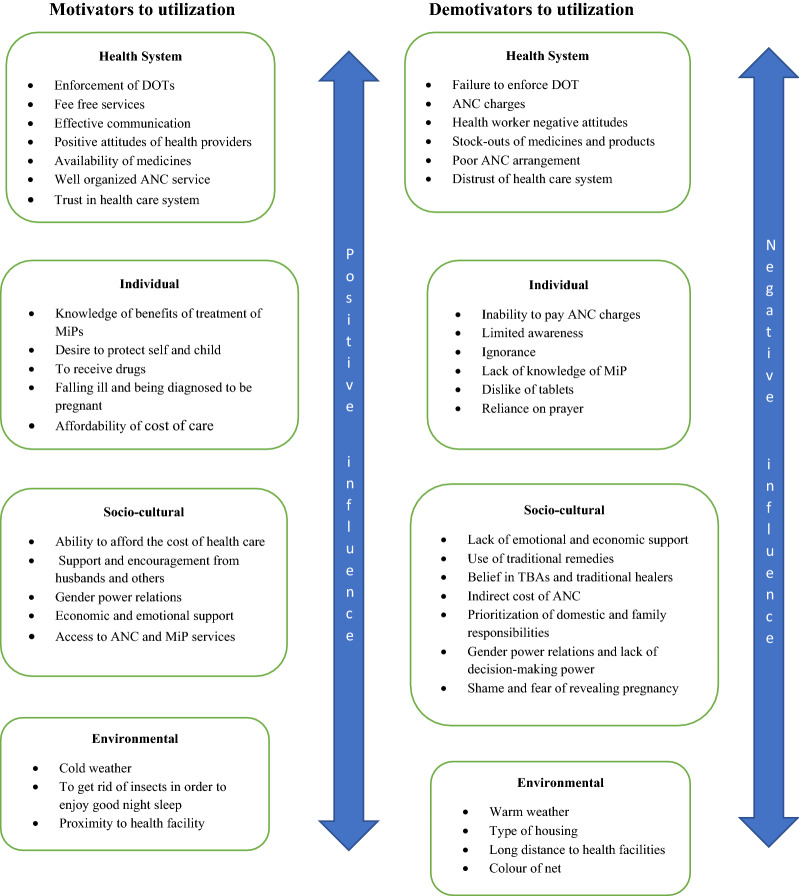
Motivation and demotivation for accessing malaria interventions

Figure 1 presents the flowchart of the search, while Tables 1 and 2 present summaries of the 27 articles that were included in the review. Factors that motivated the utilization of MiP interventions and factors that demotivated women from utilizing MiP interventions were grouped into four sub themes (health system, individual, socio-cultural and environmental. Figure 2 provides a summary of the different factors under each of the four sub-themes that can motivate pregnant women to access MiP interventions. The figure also presents the different factors under the four themes that can negatively influence pregnant women’s access to MiP interventions. The detailed results are presented in the rest of the section.

**Table 1:** Summary of studies by intervention(s) and country

WHO African region	Country	Number of studies	Type of intervention	First author(s)
Eastern Africa	Malawi	1	IPTp	Yoder et al. [47]
	Tanzania	1	IPTp and Malaria testing and treatment	Mutagonda et al. [48]
	Tanzania	1	IPTp	Mubyazi [49]
	Tanzania	1	IPTp	Mubyazi and Bloch [67]
	Uganda	2	IPTp	Rassi et al. [50] Rassi et al. [51]
	Uganda	1	LLINs	Taremwa et al. [52]
Multi country studies	Kenya, Malawi & Ghana	1	IPTp & LLINs	Pell et al. [53]
	Kenya &Mali	1	IPTp, ITNs, and Malaria testing and treatment	Hill et al. [54]
Western Africa	Ghana	3	LLINs	Aberese‑Ako et al. [17] Manu et al. [55] Quist and Adomah-Afari [56]
	Ghana	3	IPTp	Aberese-Ako et al. [57] Aberese-Ako et al. [58] Doku et al. [25]
	Mali	3	IPTp	Chukwuocha et al. [27] Hurley et al. [68] Klein et al. [59] Webster et al. [66]
	Nigeria	1	LLINs	
	Nigeria	2	IPTp & LLINs	Nyaaba et al. [3] Onyeneho et al. [60]
	Nigeria	4	IPTp	Diala et al. [24] Diala et al. [61] Onoka et al. [62] Onoka et al. [63]
Southern Africa	Mozambique	1	IPTp	Arnaldo et al. [64]
	Mozambique	1	IPTp & LLINs	Boene et al. [65]
Total		27

**Table 2:** Summary of studies included in the synthesis

First author and year	Objectives	Study population	Type of intervention	Country	Methods and analysis	Themes
Aberese‑Ako et al. [17]	To understand health system, socio‑cultural, economic and environmental dynamics in utilization of LLINs among pregnant women in two Ghanaian regions	Pregnant women Health care providers, opinion leaders	LLINs	Ghana	Ethnography using observations, informal conversations and IDIs QSR Nvivo was used to support data coding. Data were triangulated and analysed thematically	Health systems, Individual, Socio-cultural and Environmental
Aberese-Ako et al. [57]	This ethnographic study explored how health care managers dealt with existing MiP policy implementation challenges and the consequences on IPTp‑SP uptake and access to maternal health care	Health managers, health providers, pregnant women, National Health Insurance Authority officials	IPTp-SP	Ghana	Ethnography using non‑participant observations, conversations, in‑depth interviews and case studies in eight health facilities and 12 communities for 12 months in two administrative regions in Ghana. Grounded theory analysis and data coded with support of QSR Nvivo	Health system, Individual
Aberese-Ako et al. [58]	Ethnographic study explored how health system, individual and socio-cultural factors influence IPTp-SP uptake in two Ghanaian regions	Pregnant women, health providers, opinion leaders	IPTp-SP	Ghana	case studies and in-depth interviews in 8 health facilities and 8 communities in two Ghanaian regions	Health system, Individual, Socio-cultural
Arnaldo et al. [64]	Explored factors limiting access to and use of IPTp-SP rural Mozambique	46 Pregnant women 4 Health workers	IPTp-SP	Mozambique	Semi-structured interviews with pregnant women, and health workers in a rural area of southern Mozambique Data were transcribed, manually coded, and thematic analysis was done	Health system, Individual
Boene et al. [65]	To describe pregnant women’s perceptions of malaria, barriers to effective interventions and recommendations on effective interventions to prevent malaria infections	85 pregnant women	IPTp-SP, LLINs	Mozambique	Mixed methods: observations, IDIs and focused ethnographic exercises (Free-listing and Pairwise comparisons) Thematic analysis. Data from focused ethnographic exercises were summarized into frequency distribution tables and matrices	Individual
Chukwuocha et al. [27]	To assess perceptions on the use of LLINs and its implications in preventing malaria in pregnancy	Pregnant women, adolescent girls, non-pregnant women and men between 20 and 50 years old Opinion leaders, local government officials, elderly midwives, retired women leaders, drug shop owners, traditional birth attendants	LLINs	Nigeria	FGDs, IDIs key informant interviews and structured questionnaires No method of data analysis mentioned nor described in the main text	Individual, Socio-cultural
Diala et al. [61]	Examined social, cultural, and economic factors that serve as barriers to malaria treatment for pregnant women and possible factors to IPTp2 uptake in two Nigerian states	Women who had ever accessed ANC and MiP care, husbands or partners of women and health workers providing ANC and care for malaria in pregnancy	IPTp-SP	Nigeria	In-depth interviews, focus group discussions	Health system, Socio-cultural, Individual
Diala et al. [24]	Perspectives of pregnant women and ANC providers on real and perceived barriers to IPTp-SP adherence	Community-based and facility-based maternal health care providers, women of reproductive age and their husbands	IPTp-SP	Nigeria	Socio-ecological model, cross-sectional study, focus group discussions and in-depth interviews Used Atlas ti software for analysis, coded using a framework and thematic analysis	Individual, Socio-cultural, Environmental
Doku et al. [25]	Pregnant women, health workers and health managers	To investigate factors contributing to high dropout rate between IPT1 and IPT3 in the Tamale Metropolis of Ghana	IPTp-SP	Ghana	Survey, short ethnographic techniques employing IDIs and non-participant observation Ethnographic techniques and content analysis	Health system, Individual
Hill et al. [54]	Explored the delivery, access and use of interventions to control malaria in pregnancy	Non-pregnant women aged 15–49 years, pregnant women, mothers of children aged < 1 year and adolescent men	LLINs, IPTp-SP Testing and treating malaria in pregnancy	Kenya, Mali	Focus group discussions Content analysis	Health system, Socio-cultural, Individual
Hurley et al. [68]	To identify factors contributing to low uptake of intermittent preventive treatment of malaria in pregnancy with sulfadoxine-pyrimethamine (IPTp-SP)	Pregnant women	IPTp-SP	Mali	Secondary data analysis on Mali’s2012–2013 Demographic and Health Survey (DHS) IDIs, FGDs and ANC observations in six rural sites Descriptive coding supported by ATLAS.ti	Health system, Individual
Klein et al. [59]	Explored perceptions and experiences of IPTp-SP cost in Mali and its impact on uptake	Pregnant women, husbands, mothers-in-law and health workers	IPTp-SP	Mali	IDIs, FGDs, ANC observations and record reviews at health centres Topical coding supported by ATLAS.ti (version 7)	Health system Socio-cultural
Manu et al. [55]	Explored factors associated with LLIN use among pregnant women in the middle belt f Ghana	Women who had delivered six months prior to the study	LLINs	Ghana	Used IDIs and FGDs Thematic analysis of major themes put into a matrix for interpretation	Health system Individual Socio-cultural
Mubyazi [49]	Assessed knowledge, perceptions of antenatal care (ANC) services and actually delivered services and reasons for seeking ANC including intermittent presumptive treatment during pregnancy (IPTp-SP) against malaria	Pregnant women	IPTp-SP	Tanzania	Quantitative and qualitative techniques were employed, involving interviews with ANC clients, informal communications with health care workers, FGDs with mothers of young children, and intertemporal observations	Health system Individual Socio-cultural
Mubyazi and Bloch [9]	Described the experience and perceptions of pregnant women about costs and cost barriers to accessing ANC services with emphasis on IPTp-SP in rural Tanzania	Pregnant women and mothers with infants	LLINs IPTp-SP	Tanzania	FGDs and IDIs Manually coded using qualitative content analysis	Health system Environment Individual
Mutagonda et al. [48]	To assess the knowledge and awareness of pregnant women regarding the use of IPTp-SP and artemether-lumefantrine (ALu) for treatment MiP	Pregnant women	IPTp-SP Testing and treating MiP	Tanzania	Mixed method FGDs conducted with 46 pregnant women Qualitative data analysis not reported in the paper	Health system Individual
Nyaaba et al. [3]	Explored factors influencing poor uptake of IPTp-SP and use of ITNs in lower socioeconomic communities in Nigeria	Traditional birth attendants, faith-based birth attendants, health care providers	IPTp-SP LLINs	Nigeria	Semi-structured interviews with key stakeholders and focus group discussions with multi- and first-time pregnant women Thematic approach was used in data analysis with the initial coding framework generated in QRS Nvivo	Health system Socio-cultural,
Onoka et al. [62]	Examined the influence of demand side factors on IPTp-SP coverage	Women 15–49 years, who had delivered live babies during the previous year	IPTp-SP	Nigeria	FGDs Content analysis was used to generate common themes	Socio-cultural Individual
Onoka et al. [63]	Heads of maternal health units of 28 public and six private health facilities offering antenatal care (ANC) services in two districts in south-east Nigeria A checklist was used to check the availability of SP and water, review of facility staff registers to ascertain the number of health providers	Explored provider factors affecting the delivery of IPTp-SP	IPTp-SP	Nigeria	IDIs and information from checklist coded thematically. No software mentioned	Health system Individual
Onyeneho et al. [60]	Identified perceptions and attitudes towards sleeping under LLINs and uptake of recommended doses of IPTp-SP	Health workers and mothers who delivered within 6 months preceding the study, grandmothers and fathers of children born within 6 months preceding the study	LLINs IPTp-SP	Nigeria	A cross-sectional study in three local government areas. IDIs and FGDs Thematic coding and Atlas.ti software was used in managing the data	Health system Individual
Pell et al. [53]	Provided insight into the social and cultural context to the uptake of interventions for malaria prevention and control in four sites within three countries	Pregnant women with pregnant women, their relatives, opinion leaders, other community members and health providers	LLINs IPTp-SP	Ghana, Kenya, Malawi	IDIs and group interviews. Observations at health facilities and in local communities Atlas.ti was used to support coding	Health system Socio-cultural Individual Environmental
Quist and Adomah Afari [56]	Explore how socio-cultural beliefs and practices influence knowledge, attitude and perception of LLIN use in the control of malaria amongst pregnant women attending antenatal clinic	Pregnant women	LLINs	Ghana	Interviews and documentary review NVivo, framework analysis was applied to classify emerging themes and the findings interpreted using the health belief model	Individual
Rassi et al. [50]	Assessed demand side barriers to accessibility, affordability and acceptability of IPTp-SP intervention	District health officials, health workers, women who attended antenatal care and opinion leaders	IPTp-SP	Uganda	IDIs, Thematic analysis	Individual Socio-cultural Environmental
Rassi et al. [51]	This study assessed supply-side barriers (health service provider), which impede IPTp-SP uptake in Uganda especially among women who attend ANC	District health officials, health workers, women attending antenatal care and opinion leaders	IPTp-SP	Uganda	Document and record review in four health centres IDIs Used QSR NVivo to support thematic data coding and analysis	Health system
Taremwa et al. [52]	Explored knowledge, attitude, and behaviour towards the use of LLINs as a nightly malaria prevention strategy for pregnant women and children	Pregnant women and caregivers of children under five years old. Local council leaders, district health inspectors, religious leaders, health workers and members of village health teams (VHTs)	LLINs	Uganda	Mixed methods Qualitative aspect involved conducting key informant interviews Thematic content analysis. Manually analysed	Socio-cultural Individual
Webster et al. [66]	To explain quantitative data from a related study which identified ineffective processes in the delivery of IPTp-SP and LLINS in one district in Mali	Health workers at the national, regional, district and health facility levels	IPTp-SP	Mali	In-depth interviews with health workers at the national, regional, district and health facility levels Thematic coding using content analysis	Health system
Yoder et al. [47]	Examined the experiences of nurses and midwives in providing antenatal care (ANC) services	Health care providers	IPTp-SP	Malawi	Interviews with a semi-structured interview guide Content analysis	Health system

### Motivation in MiP intervention utilization

Factors that positively influenced pregnant women or enabled them to access testing and treatment for malaria, access to or use of LLINs or facilitated access to IPTp-SP were identified as motivators. They were categorized under one of the four sub-themes: health system, individual, socio-cultural and environmental, which have been presented in the subsequent sub sections.

### Health system factors motivating utilization of MiP care

Several studies reported that well organized ANC procedures and the enforcement of directly observed therapy (DOT) motivated women to take IPTp-SP. It was further noted that in such situations even women who complained of side-effects still took it [47, 58, 65, 66]. The provision of drugs, LLINs and health workers exhibiting positive attitudes motivated women to attend ANC regularly. Such women also encouraged others to begin ANC early in Nigeria [24, 61] and Uganda [50, 51]. However, the stated studies noted that once women took LLINs or if there was a shortage of LLINs, women stopped attending ANC [24, 50, 51]. Also, the free distribution of IPTp and LLINs to women motivated uptake in Ghana, Mozambique and Nigeria [3, 17, 57, 64].

Webster et al. [66], Yoder et al. [47] and Aberese-Ako et al. [58] reported that sensitizing and encouraging women on the need to take IPTp and making water available to them at the ANC motivated women to take SP. When women received explanation on the relevance of taking SP and other medications from health workers, they became motivated to take them, even for those who experienced mild side-effects and would otherwise have refused to take SP and other drugs [50]. Additionally, effective communication with health providers regarding the next scheduled visit and the number of doses of IPTp-SP required motivated some women to honor scheduled visits to enable them to take all the required doses [58].

Trust in the health care system compared to chemists and other sources motivated women to take IPTp-SP, even when complaining of side effects [65]. Women’s high trust in health care providers and health facilities motivated them to take their anti-malarial treatment even when they were not sure of the dosage and did not like the bitter taste [24, 50, 62]. Some women had experienced positive effects of regular visits to the ANC in previous pregnancies, resulting in the building of trust in the health care system, which motivated them to return to the ANC in subsequent pregnancies as well as adhere to all the care offered them [57].

### Individual factors motivating attendance to ANC and utilization of MiP interventions

The key motivation for women to attend ANC, was to have their pregnancy monitored and managed with the hope that it will lead to healthier pregnancies and babies and reduce the risk of complications at delivery [3, 24, 49, 50, 54, 64, 67]. A respondent in an interview stated: “*… because I felt I must take care of myself and the baby to ensure that both of us are healthy”* [50:7]. Other reasons for ANC attendance was to avoid reprimand, since women who did not attend ANC at least once risked being sent away by midwives, even if they came with complications at delivery [24, 49, 54, 67].

Other motivations for attending ANC were to receive drugs including IPTp-SP and to have laboratory tests done for HIV and malaria [24, 64]. Respondents perceived that preventing the onset of malaria saved money compared to treatment of the illness once it had progressed [54]. Mubyazi [49], reported that in Tanzania women who attended ANC were motivated by the desire to receive a price discounted LLIN voucher early, to enable them to protect themselves against mosquito bites, which could result in malaria.

Onoka et al. [63], Nyaaba et al. [3] and Aberese-Ako et al. [58] noted that women who were personally motivated or had knowledge on the effects of malaria in pregnancy did not mind the price of IPTp-SP and malarial treatment drugs. Such women were always ready to take it, because they believed that it was good for them and their babies. Arnaldo et al., [64] also reported that women felt the drugs were good for them and their babies. Additionally, Aberese-Ako et al. [58] reported that women who were gainfully employed accessed MiP interventions.

Manu et al. [55] and Quist and Adomah-Afari [56] noted that knowing that LLINs protected the mother and the unborn baby from malaria and other health problems associated with non-use, motivated use in Ghana. Nyaaba et al. [3], found that a high perception of the seriousness of the effect of malaria in pregnancy, belief that usage of LLINs was beneficial to mothers and children through the prevention of malaria and a high awareness that the prevention of malaria was a cheaper option than treatment motivated women to use it. Taremwa et al. [52] and Quist and Adomah-Afari [56] found that motivation for using LLINs was based on women’s personal experiences and community members’ experiences of the positive effect of using LLINs.

### Socio-cultural factors motivating women

Gender power relations within the household played an important role in women's utilization of maternal health care services. This is because, husbands made decisions on whether wives should seek medical care [24]. Additionally, husbands encouraged wives to access ANC and MiP interventions. Some husbands felt it was their duty to exercise power over their wives by compelling or encouraging them to commence ANC attendance early, to seek health care and to comply with prescribed medications as well as to use LLINs regularly [17, 24, 61, 67].

Husbands who knew that their wives could obtain treatment from the health facility encouraged them to attend ANC and offered them financial support to enable them pay for transport and other ANC charges [24, 57]. Husbands encouraged their wives to take their medications, reminded them to honour scheduled ANC visits and others helped to monitor their wives’ health. Some husbands also accompanied their wives to the ANC or asked a female relative to accompany them. Other forms of support indirectly linked to malaria treatment and prevention in pregnancy were husbands supporting their wives to perform household chores and ensuring that they ate traditionally known healthy diets [24].

Other socio-cultural factors that motivated the uptake of MiP interventions were support and encouragement from relatives and friends, the experiences of relatives attending ANC and using LLINs with positive effects and availability of social support from friends and relatives. Sometimes other family members and friends also motivated women to access ANC and MiP services [24, 48, 68]. Close and trusted relatives, friends and neighbour supported pregnant women to visit health facilities, encouraged them to honour ANC appointments and to seek medical help when they were ill [24, 57]. Mother in-laws were very supportive in helping women to negotiate with their husbands to obtain money to access health care in Mali [59].

Also, friends served as social support and interactions between them, and influenced decisions to seek care and to manage ailments [24, 57]. A respondent shared her source of motivation in an interview:

“*A friend came to visit me. She saw how terribly ill I was. After two days, she came to visit me and she saw my condition had not improved. Therefore, she told me to seek medical attention or she will never visit me again. So, to me she was caring; that was why she advised me*”.

Some friends also accompanied their pregnant friends to the ANC and those who attended ANC provided collective support by sharing of experiences, making new friends at the facilities and encouraging each other to take their medications such as malaria drugs [24, 61]. Women were influenced to use LLINs if they observed in their households that others had benefitted from using them. For instance, pregnant women were motivated to use LLINs if the entire household had not experienced malaria since initiating LLIN use [17, 52].

### Environmental factors motivating access of MiP

LLINs were reported to be used more in the rainy season than in the dry season [27, 54]. In rural Mali, it was noted that everyone, including pregnant women slept under LLINs in order to wade off mosquitoes and to enjoy good night sleep [54]. Manu et al. [55] and Quist and Adomah-Afari [56] equally reported that women were motivated to use LLINs, because they were aware that it helped to wade off mosquitoes and other insects, which ensured that they had a good night’s sleep.

Women who lived in areas close to health facilities were motivated to access ANC care, because they did not have to incur transport costs [9]. Such women accessed maternal health care services including MiP interventions, receiving the optimum doses of IPTp-SP and LLINs on a timely basis.

### Demotivation

Factors that contributed negatively or discouraged women from accessing MiP interventions were defined as demotivators. They were categorized under the four sub-themes: health system, individual, socio-cultural and environmental, which have been presented in the sub-sections below.

### Health system factors

Stock-out of SP at the national level affected its supply and distribution in health facilities, which demotivated women from accessing ANC service [3, 48, 57, 63]. Stock-outs of SP in health facilities led to health workers prescribing IPTp-SP for women to buy from private pharmacies, contributing to the burden of cost and increase in travel distance for ANC services [3, 57, 63, 68]. In Ghana and Nigeria, health workers admitted that they could not guarantee that women actually bought and took the prescription, since they could not apply DOT in such situations [3, 57]. Another challenge anticipated in Nigeria concerning stock-outs was the fear that women could buy IPTp-SP from unregulated sources [3].

Nyaaba et al. [3], reported that women who attended ANC at private facilities or accessed the services of traditional birth attendants (TBAs) did not receive IPTp-SP and LLINs. Some women could not pay for some ANC services provided at health facilities. Also, poor women could not afford the cost of subsidized LLINs and transportation to the distributing centres [48, 49, 51], which denied them the opportunity to access IPTp-SP, LLINs and other maternal health services. Hurley et al. [68], Aberese-Ako et al. [57] and Nyaaba et al. [3] reported that while the national policy directed that IPTp-SP should be given free of charge, sometimes, facilities instituted charges for IPTp-SP, which affected poor women’s ability to access them. Hurley et al. [68] also reported that women were demotivated from accessing SP in Mali, because, though SP was free, it was usually added to a long and costly list of prescriptions. Women could not distinguish between the free SP from the long list, so they perceived that ANC was costly and thus, sometimes left the ANC without accessing it. Some facilities charged fees for testing for malaria in pregnancy, which demotivated poor women from accessing MiP services [57].

The shortage of health providers to administer ANC care to women contributed to women’s limited access to ANC and MiP intervention services [48, 63]. Other studies found that the limited number of health care providers resulted in few workers attending to high patient load, which demotivated health providers from providing quality services [48, 63, 64]. Health providers reported that they coped with huge number of patients by offering quick consultation to women without any explanation about the purpose for taking SP.

A nurse providing maternal health service stated: *“Usually*, *we are overworked*, *with much to do*... *and when the women come for a prenatal* *consultation*, *I often just give the tablets for malaria prevention*, *sometimes even without.* *explaining carefully the details”.* Thus, women were not well informed of the benefits of MiP interventions, which affected uptake. Another consequence of the high patient load was long queues at the ANC leading to long waiting times, which demotivated women from accessing IPTp-SP and other maternal health care services [63, 64].

Whilst women who experienced side effects from previous doses of SP were not given IPTp-SP in some facilities [50, 63], others refused to take SP, because they previously experienced side effects as a result of taking it on empty stomachs. Some health workers were compelled to apply DOT to ensure that such women took it [48, 58]. Hurley et al. [68] and Onoka et al. [63] reported that the misperception that SP should not be taken on an empty stomach, contributed to health workers giving SP to women to go home to take after meals. However, this decision did not guarantee uptake at home.

Onoka et al. [63], found that all the health workers who participated in their study knew the correct drug for IPTp-SP, however majority of them did not know that IPTp-SP was supposed to be given in the second and third trimesters and under DOTs. Health providers gave varied views on the timing of administration of IPTp-SP and only half of them knew the correct strategy for administering IPTp-SP under DOTs. Nyaaba et al. [3] on the other hand, reported that very few health workers enforced DOT. Also, sometimes health workers failed to detect early pregnancy, which prevented them from putting women on IPTp-SP and other ANC services early [49].

Studies in Kenya, Ghana [53, 58], Mozambique [64] and Nigeria [63], indicated that when health workers gave little information or no education to women on IPTp-SP and DOT was not practised, women were not motivated to take SP. Nyaaba et al. [3] found that women when offered IPTp-SP without explanation to take at home did not take the tablets, because they assumed that they were already taking enough drugs. A pregnant woman explained: “*I use pregnant care [vitamin supplements]. So I*’*ll collect it [SP] and leave it at home*... *they don*’*t say anything about the drugs, so all the drugs given to me are already in pregnant care, which is 2-in-1*...*”*[3:8]. Mubyazi [49] and Nyaaba et al. [3] on the other hand noted that women were not motivated to take SP, because the concept of IPTp-SP as a presumptive treatment and not for treatment of malaria was not clear to women. Women raised concerns about taking drugs during pregnancy when they were not sick. Nyaaba et al. [3] also noted that the perception that a pregnant woman should not take drugs when she is not sick demotivated women from taking SP.

Shortage of SP demotivated women from attending ANC and contributed to IPTp-SP dropout rates in Ghana, Nigeria and Tanzania [24, 25, 48, 61]. Additionally, the lack of educational materials such as pamphlets or pictures on MiP to facilitate teaching women about the benefits of IPTp-SP contributed to the lack of knowledge, which did not motivate women to take SP. A maternal and child nurse stated: *“Perhaps, if we had some illustrative pictures or.*

*images of a person who did not complete [the] treatment*, *it would be helpful*…*, because some do not complete the recommended dosage, because they think the tablets are strong”* [62:7].

A few providers indicated that the failure of their facilities to provide water to women to facilitate the implementation of DOTs demotivated women from taking IPTp-SP [63]. Negative health worker attitudes in ANC clinics demotivated some women from commencing ANC attendance early, resulting in the late uptake of IPTp-SP and some not receiving LLINs early during pregnancy [3, 9, 24, 25, 27, 49, 60, 67]. In some facilities in Nigeria, pregnant women were required to go to the clinic with their husbands, which delayed clinic attendance and affected the timely provision of IPTp-SP [24, 48].

### Individual factors demotivating women from accessing MiP interventions

Difficulties in swallowing tablets, nausea and other side effects such as sweating, drowsiness and frequent urination were commonly reported by pregnant women as key reasons demotivating them from taking IPTp-SP [3]. Some women indicated a preference for injections and others said they will prefer that the drug will be put in liquid form, as they found it difficult to swallow the tablets [3, 58]. Aberese-Ako et al. [58], Mubyazi and Bloch [67] and Nyaaba et al. [3] reported that some women deliberately delayed or postponed ANC attendance for fear of the belief that SP could have side-effects on the baby. This attitude was more common among clients with higher levels of education such as teachers.

Poor knowledge on how and when to use LLINs and low awareness of IPTp-SP did not motivate women to access IPTp-SP [3, 57, 65]. Some women preferred to use spiritual means such as visiting prayer camps, using spiritual water, fasting based on their pastors’ instructions or taking herbs to protect themselves from spiritual attacks and curses that they could be afflicted with during pregnancy [3, 57, 58]. Onoka et al. [62] reported in their study that, in rural facilities, IPTp-SP was given to women to take home. However, those who were taking herbal drugs, kept the SP and delayed taking it and rather completed taking the herbal treatment before taking it.

Some women complained of discomfort, while others experienced difficulty breathing whenever they used LLINs [27, 55, 60, 65]. Some also complained that they reacted to the chemicals used to treat the net, which they described as having an unpleasant smell that made them vomit. Others also complained of heat from the chemicals used in treating the LLINs, developing rashes and itching as reasons demotivating them from using LLINs [3, 17]. Other factors that demotivated women from using LLINs included ignorance, lack of access and poverty [17, 27, 54]. Some women who owned LLINs but did not use them complained that they were too small in size to accommodate them and their children [55]. Hill et al. [54] reported that adolescents were not motivated to use LLINs, as they explained that it was not their habit. Another reason was feeling of laziness to regularly hang the net at night.

The fear of needle pricks or being tested for HIV/AIDS demotivated some women, especially adolescents and young women, from attending ANC. This resulted in missed opportunities to receive information on malaria, LLINs and IPTp-SP [50]. Some delayed ANC attendance till the pregnancy was in an advanced stage, thus missing out on early use of LLINs and the chance to receive the recommended three doses of IPTp-SP [48, 64]. A nurse providing maternal and child health care services explained in an interview: "*Women come for the first ANC visit at thirteen weeks of pregnancy and may receive up to three doses*, *but in most of the cases women… first visit at eight months*. *These will not complete the recommended doses*" [64:7].

Self-medication was particularly common with uninsured women (to avoid the cost of both transport and medical care), and on other occasions for cases of mild malaria some combined self-medication with hospital treatment [53]. Boene et al. [65] and Arnaldo et al. [64] in their studies in Mozambique, reported that malaria was not viewed as a threat to pregnancy and study respondents did not know that malaria infection could have associated adverse maternal and birth outcomes. Consequently, they were not motivated to access MiP care.

### Socio-cultural factors demotivating women from accessing MiP interventions

It was noted that poor women especially those who did not receive financial support from their husbands and close relatives, could not afford the cost relating to transport to accredited stores or shops to buy LLINs or LLIN vouchers. Additionally, they could not afford to pay for IPTp-SP, malaria treatment and ANC services [9, 24, 27, 49, 54, 55, 59, 68].

A study respondent stated: “*Some of our husbands have no money to give us. Some are very poor, and one cannot go to the hospital, even government hospitals, without money. So, your only option is to stay at home with malaria and have faith in God to cure you*”. This resulted in non-attendance, late attendance and irregular attendance to the ANC, which resulted in late or non-uptake of IPTp-SP [9, 59, 68]. A woman stated in an interview: “*We pay for every type of drug here, so the government has to look at this problem closely. Okay, we are blamed for coming late to the clinic, but what do they expect, if we have no money?”*.

Other indirect costs such as the inability to afford a maternity dress resulted in late ANC attendance and irregular attendance [51]. Moreover, sometimes women did not return to the ANC, because they could not afford the nutritional diet that was prescribed for them at the ANC [51]. Additionally, women prioritized their responsibilities and work over accessing preventive care. For instance, women sometimes failed to attend ANC to facilitate IPTp-SP uptake. Some also missed scheduled ANC visits due to prioritizing domestic work, taking care of children and farming activities during the farming season [48, 64, 67].

In Mali women had to seek permission from their husbands and male heads of families to attend ANC [59]. Additionally, women indicated that husbands and family heads made key financial decisions on health care, which affected women’s access to ANC care [54, 59]. Some husbands, as reported in studies conducted in Nigeria [24] and Mali [59], did not encourage their wives to go to the health facility for ANC service. This affected timing and the regularity of accessing maternal health care services. Some women had to negotiate with their husbands or other male relatives to get money for ANC. Therefore, on occasions when they were unsuccessful, they had to borrow money or missed out on ANC attendance [59]. Women who faced difficulty in finding alternative sources of money to pay for ANC reduced the frequency of ANC visits [54, 59]. In Mali, pregnant women who depended on their husbands for money to access health care were reluctant to disclose ailments to their husbands, because, the cost involved was thought to be irritating to their husbands [54]. The societal perception that women are not supposed to have money demotivated women from buying and using LLINs in Nigeria, even though they knew the benefits of LLINs. They feared that if they used their own money to buy LLINs, their husbands would question them over the suspicion that they got the money from other men [27].

Other socio-cultural factors that demotivated women from attending ANC early were shame and fear that if they revealed their pregnancy early, they could be perceived as being boastful, hence, eliciting envy, jealousy, social exclusion and bewitchment [49, 68]. In Kenya, the shame of being pregnant out of wedlock or being pregnant at the same time as their mothers and thus having to share clinic space discouraged adolescents and younger women from attending ANC [54]. Late recognition of pregnancy especially among young women and adolescents contributed to late visits to the ANC, which affected early initiation of IPTp-SP and the number of IPTp-SP doses that some took by the time of delivery [49].

Community members, husbands, mothers and mothers-in-law held strong beliefs that traditional healers and traditional birth attendants (TBAs) had better knowledge of medicines for treating malaria than health workers, which motivated women to seek health care from them rather than visiting a health facility [24, 27, 60, 67]. A respondent stated in an interview:

*“As with husbands, some parents do not encourage their daughters to go to a hospital during pregnancy, because of their traditional beliefs and recommend traditional birth attendants, herbalists or native doctors rather than health care providers in government health facilities*”.

Studies from Nigeria suggest that some husbands and older women believe that their parents did not attend ANC, that male health workers will attend to their wives and others perceive that health workers are not more knowledgeable than traditional healers [24, 60]. Such perceptions contributed to the belief that pregnant women did not need to attend ANC or to seek treatment for malaria at the hospital [24, 60].

Health shopping was common in a study in Nigeria [60]. Pregnant women who were afflicted with malaria first visited the drug store to buy recommended drugs from the chemist [3, 60]. They only went to the hospital when the purchased drugs did not work [60]. A husband stated in an FGD:

“…*whenever she is sick, we go to the chemist first and buy some drugs. If there is any dealer there, we ask him what drug to buy. If not, we buy the one we know. If that does not work, we shall now go to [the] nurse (auxiliary nurse) to give her an injection. When we have tried all these ones and it doesn’t work, we will now go to [the] hospital”*.

Community members and pregnant women believed that malaria that persisted or was severe could only be treated by using herbal remedies, boiling leaves or visiting the traditional herbalist [24, 53, 60]. Many participants confirmed that seeking treatment in modern health facilities was generally viewed as a last resort; usually when the disease posed a major threat to life [24, 53]. Other reasons for using traditional medicine was the belief that it works faster, it is cheaper and more accessible [24, 61]. Some women used traditional remedies consisting of boiled leaves of pawpaw, mango and ginger as key strategies to preventing malaria in pregnancy, while others preferred to visit TBAs and faith-based attendants instead of attending ANC services [3, 60]. Quist and Adomah-Afari [56] and Aberese‑Ako, Magnussen [17], found that some community members burn herbs, orange peels and dried palm-nut with the aim that the scent emitting from the smoke would drive away mosquitoes and, therefore, did not use LLINs. However, respondents admitted that it only works for a short time.

There were misconceptions among study participants, which demotivated pregnant women from using LLINs [27, 52, 56]. In Ghana it was believed that the chemicals used in treating the net could cause abortion [56], in Uganda its use was associated with cancer [52], while in Nigeria it was believed that it could affect one’s ability to breathe, and it was also perceived as poisonous and could kill [27]. A respondent in an FGD stated: “*We fear we may die because these chemicals are poisonous*”. Aberese‑Ako et al. [17] Pell et al. [53] and Taremwa et al. [52] observed that LLINs were used for other purposes such as netting for gardens, fishing nets, latrine doors, crop protectors, to raise chickens and as decorative wall hangings.

### Environmental factors

Warm weather demotivated pregnant women from using LLINs. They complained of heat, especially in the warm season, feeling hot, uncomfortable, sweating and others said they experienced difficulty in breathing when they used LLINs [17, 27, 54, 55, 60]. Distance to health facilities coupled with lack of access to transport in Mali [68] and long distances to health care facilities in Tanzania [48] and in Nigeria [3], demotivated women and contributed to poor ANC attendance, which impacted on women’s access to MiP services.

Long distances to heath facilities resulting in women getting there late and risking being turned away or asked to come at another time frustrated them [9, 24]. Such women attended ANC irregularly, which impacted on regular uptake of IPTp-SP [9, 24]. Mubyazi and Bloch [9], noted that women living in rural areas of Tanzania found the distance to health facilities more challenging than women living in urban areas, where there are better access roads and transport service. In the case of Nigeria, women in peri-urban areas complained about long distances to tertiary facilities, the time taken for the trip, and high transport fares [24]. Also, a combination of long travel distances and waiting times at clinics demotivated women from early ANC registration in Tanzania [9]. Women who lived far from health facilities were the least motivated, because it contributed to delays in starting ANC early, which reduced the likelihood of them receiving LLINs early and completing the required dose of IPTp-SP by the time of delivery. A woman who attended her first ANC at six months of gestation explained:

*" This [waiting time] makes it a bit difficult, because I live far away from the health centre. When I imagine that I will stay here for a long time I give up"* [pregnant woman].

In Ghana, some women were not motivated to use LLINs, because the nets were white and not suitable for their mud homes. They easily became dirty when they hanged them over bed linens which were placed on the bare floor [55]. Others reported that they did not use their nets, because the architectural design of their rooms made it difficult to hang them and poor ventilation made it difficult to use them [17, 55, 56].

## Discussion

This was a meta-ethnographic synthesis of 27 qualitative research articles on the WHO recommended MiP interventions (LLINs, IPTp-SP and testing and treating malaria), for malaria endemic countries in sub-Saharan Africa. The results suggest that health system, individual, socio-cultural and environmental factors, influenced the uptake of MiP interventions.

The study found that health system factors that motivated women to access MiP interventions are diverse and included trust in the health care system, positive attitude of health providers, availability of health care providers, enforcement of DOT, well organized ANC service delivery, effective communication to ANC attendees and fee-free maternal health care services. Similarly, Exavery et al. [69], reported that women who received information and counselling at the ANC were more likely to complete optimal doses of IPTp-SP. In addition, other studies have noted that the enforcement of DOT and availability of water, positive attitude of health workers, trust in the health care system and effective communication on MiP interventions to women positively impacted on women’s access to MiP interventions [36, 70, 71]. It is important that facilities maintain and strengthen health system factors that motivate women to utilize MiP services. Considering that most sub-Saharan African countries have large populations of women who are below the poverty line, it is crucial that maternal health care services are provided at a much-subsidized rate in order to motivate them to use them.

Health system factors such as failure to enforce DOTs, charges for ANC and IPTp-SP services, negative attitudes of maternal health providers, stock-outs and poor ANC arrangements demotivated pregnant women from accessing ANC and MiP services. In addition, failure to provide women with information on the benefits of MiP interventions, shortage of health staff, resulting in high patient load and failure to offer women SP due to complaints of side effects affected uptake of MiP interventions and were reported in some of the analysed studies. Other studies have equally reported that health system barriers such as stockouts and user fees impacted negatively on women’s access to IPTp-SP and LLIN in sub-Saharan Africa [28, 33, 34, 36, 72]. Another study has shown that women who worried about the lack of health care providers at health facilities were not likely to complete optimal doses of SP [73]. This is in line with a review study on maternal health that noted that poor reimbursement from the government contributed to charges in health facilities and poor quality maternal health care in the sub-Saharan African region [74]. It is, therefore, very important that health system gaps are addressed, in order to motivate women to access health care. Improving frontline health worker attitudes and providing them with the needed medical supplies will contribute to improving maternal health care as well as improve women’s access to MiP interventions. There is a need to boost ANC staff strength and to ensure effective organization of ANC. At the national level, it is important that, there is the political will to allocate adequate funds for maternal health care and to strengthen the channel of supply and distribution of SP, RDTs and LLINs from the national to the district and facility levels to reach the last mile.

Individual factors such as knowledge of the relevance of MiP interventions, desire to protect oneself and the unborn baby, to receive drugs and the ability to afford the cost of ANC motivated women to access MiP interventions. In addition, the desire to prevent malaria infection, women and family members’ experiences of the positive effect of using LLINs also motivated women. It transpires across studies that women who have knowledge of the negative consequences of malaria in pregnancy are more likely to visit the ANC frequently and to complete higher doses of SP compared to those who do not have knowledge and this also applies in terms of women who have knowledge of the effects of MiP on pregnancy [33, 73, 75]. There is the need that women are given ample education on the relevance of MiP interventions, to enhance the acceptance, use and adherence to MiP interventions.

In a number of studies, it was found that poor ANC attendance, the concept of IPTp-SP as a presumptive treatment and reliance on prayer contributed to women not accessing malarial preventive interventions. Similarly, other studies have reported that low knowledge of malaria and malaria interventions served as barriers to accessing treatment for malaria among pregnant women, the use of IPTp-SP and LLINs in malaria endemic countries [23, 33, 34]. ANC education and encouraging peer education among ANC attendants could facilitate the utilization of MiP interventions.

The ability to afford the cost of health care and economic and emotional support and encouragement from husbands, family members and friends motivated women to access ANC and MiP services. Other motivators for accessing MiP interventions were receiving information from husbands and friends on the availability of MiP services and women observing that relatives who used LLINs did not fall sick of malaria. Other studies have also found that financial and emotional support from partners, family and friends influenced women’s utilization of MiP interventions. This is in line with findings from other studies in sub-Saharan Africa [76–78]. In addition, Orobaton et al. [79] and Okeibunor et al. [80] found that community-based programmes, such as health education, distribution of IPTp-SP and home based management of malaria increase acceptance and uptake of LLINs, IPTp-SP and treatment of malaria in children and pregnant women. WHO places emphasis on the strengthening of community health worker programmes for HIV, TB and malaria services and community involvement in health care interventions, which could ensure a well-informed community that can support pregnant women to use maternal health services.

Poverty and the financial cost of antenatal and delivery care demotivated women from utilizing health services, which affected their ability to access MiP interventions. This included inability to afford a maternity dress and the nutritional diet recommended at the ANC and the opportunity cost of leaving domestic tasks to access preventive care. Fear of revealing pregnancy early, which could invite spiritual attacks and visiting other sources in order to prevent spiritual attacks during pregnancy contributed to delays in accessing MiP interventions. Women’s lack of power and having to seek permission from husbands and male heads of families to attend ANC, demotivated women from utilizing MiP interventions. A study by Ameyaw et al. [81] in 20 sub-Saharan African countries found that community level factors such as poverty and ignorance of the relevance of IPTp-SP negatively impacted on IPTp-SP uptake. Other studies have noted gender power relations, women’s lack of decision-making power and other cultural practices negatively impacting on women’s access to MiP interventions [36, 82]. In addition, Konje et al. [83] and Jaiteh et al. [84] reported that socio-cultural beliefs and poverty prevented women from accessing maternal health care and HIV/AIDS services in health facilities. It is important that policies ensure that maternal health care is largely subsidized in order to make it accessible to poor women and the formal health care sector needs to strengthen community engagement in order to ensure that superstitious beliefs are overcome to facilitate women accessing maternal health care, including MiP interventions.

Pregnant adolescents, who are at higher risk of malaria infection and its concomitant effects, were the least motivated to access MiP interventions early and regularly, because most of them are unemployed, receive little support from the men who made them pregnant, their lower position in society and stigmatization. Other studies have reported on adolescents keeping their pregnancy hidden especially during the early stages due to shyness, shame and the fear of being bewitched [84, 85]. Thus, community involvement through education, outreach programmes and mobilization for maternal health care is crucial and urgent for effective MiP interventions. Establishing special clinics for adolescents, could encourage and promote adolescents’ utilization of MiP and other maternal health interventions.

Environmental factors such as proximity to a health facility, the rainy season, the existence of insects and the cold season motivated uptake of MiP interventions especially the use of LLINs. Other studies have equally found that nearness to a health facility motivated access to IPTp-SP and LLINs, whiles perceived comfort of sleeping under an LLIN motivated use [82, 86]. This suggests that environmental factors need to be taken into consideration in designing MiP interventions. Ghana has recently extended IPTp-SP services to communities through the Community-Based Health Planning and Services (CHPS) programme in order to increase uptake [87]. This is commendable and could be replicated in other sub-Saharan African countries, as such an approach could facilitate uptake of IPTp-SP in communities, where women who would otherwise skip regular ANC attendance due to long distances, the cost of transport and other ANC charges.

Environmental factors such as warm weather and long distance to health facilities especially for women in rural areas who struggled to access services in distant locations demotivated women from accessing MiP interventions. The design of houses and construction makes it difficult to utilize LLINs. Azizi et al. [73] corroborate the review finding that women who experienced long distances to health facilities and lacked transport to visit facilities did not attend ANC regularly and were not likely to complete the optimum doses of IPTp-SP. In addition, Bhutta et al. [88] have noted that major barriers to effective uptake of antenatal and intrapartum services in low-resource settings include cost, distance and the time needed to access health care. This has implication for effective delivery of MiP services. Boosting the staff strength in health facilities will ensure that women who travel over long distances receive prompt treatment when they get to health facilities.

### Study limitation

Only articles written in English were considered in this review, thus limiting information on articles written in French and Portuguese, which would have been more representative of the sub-region.

## Conclusion

This meta-ethnographic review shows that multiple factors including health system, environmental, socio-cultural and individual influence the utilization of MiP interventions. Consequently, it is important that the different factors are taken into consideration in MiP interventions efforts to facilitate access and uptake.

Governments in sub-Sahara Africa need to strengthen maternal health service delivery through free health care delivery, boosting health care staff numbers and communicating effectively with beneficiary communities to understand the package of interventions and those that are free. It is important that national health care systems ensure adequate numbers of health care providers are posted to ANC facilities to ensure a balance of health worker numbers in proportion to patient load.

There is the need to strengthen health system-community engagement in order to inform and involve communities in health care delivery including MiP interventions. Community engagement could facilitate community understanding and appreciation of MiP interventions and garner support for pregnant women in their respective communities. Also, it could help pregnant women to acquire more knowledge on the effect of malaria in pregnancy, which could promote acceptance and utilization of MiP interventions.

## Data Availability

The datasets are published articles that have all been referenced in this paper and are available on the internet.

## Footnotes

1 In 2000 African leaders met in Abuja under the Roll Back malaria Partnership and set targets that 60% of pregnant women would have access antimalarial chemoprophylaxis or intermittent preventive treatment (IPTp) and 80% use of insecticide-treated nets by 2005. Subsequently, more ambitious targets were reset to 100 % use of both interventions by 2015 [15].
